# Supplementary material for: Empirical methods for controlling false positives and estimating confidence in ChIP-Seq peaks
Source: BMC Bioinformatics. 2008 Dec 5;9:523. doi: 10.1186/1471-2105-9-523 (PMC2628906; doi:10.1186/1471-2105-9-523)
Supplement: Additional File 1 — A variety of html documents from the USeq web site detailing the available applications, their best usage, output file type descriptions, command line menus, etc. [file 1471-2105-9-523-S1.zip › USeqUserGuides/JavaDoc.zip!/JavaDoc/util/apps/GeneCentricCNVParser.html]

GeneCentricCNVParser


|  |  |  |
| --- | --- | --- |
| **Overview** \| **Package** \| **Class** \| **Use** \| **Tree** \| **Deprecated** \| **Index** \| **Help** | |  |
| **PREV CLASS**   **NEXT CLASS** | **FRAMES**    **NO FRAMES**     **All Classes** |  |
| SUMMARY: NESTED \| FIELD \| CONSTR \| METHOD | DETAIL: FIELD \| CONSTR \| METHOD |  |


---


## util.apps Class GeneCentricCNVParser

```
java.lang.Object
  util.apps.GeneCentricCNVParser
```

---

``` public class GeneCentricCNVParser extends java.lang.Object ```

---

**Constructor Summary**

`GeneCentricCNVParser(java.lang.String[] args)`


| **Method Summary** | |
| --- | --- |
| `void` | `loadScores(java.io.File[] files, java.lang.String dataset)` |
| `static void` | `main(java.lang.String[] args)` |
| `void` | `printResults()` |
| `void` | `setScores()` |

**Methods inherited from class java.lang.Object**

`equals, getClass, hashCode, notify, notifyAll, toString, wait, wait, wait`

**Constructor Detail**

### GeneCentricCNVParser

```
public GeneCentricCNVParser(java.lang.String[] args)
```


**Method Detail**

### printResults

```
public void printResults()
```

---


### setScores

```
public void setScores()
```

---


### loadScores

```
public void loadScores(java.io.File[] files,
                       java.lang.String dataset)
```

---


### main

```
public static void main(java.lang.String[] args)
```


---


|  |  |  |
| --- | --- | --- |
| **Overview** \| **Package** \| **Class** \| **Use** \| **Tree** \| **Deprecated** \| **Index** \| **Help** | |  |
| **PREV CLASS**   **NEXT CLASS** | **FRAMES**    **NO FRAMES**     **All Classes** |  |
| SUMMARY: NESTED \| FIELD \| CONSTR \| METHOD | DETAIL: FIELD \| CONSTR \| METHOD |  |


---
